# Simple and rapid detection of common fetal aneuploidies using peptide nucleic acid probe-based real-time polymerase chain reaction

**DOI:** 10.1038/s41598-021-02507-5

**Published:** 2022-01-07

**Authors:** Subeen Hong, Seung Mi Lee, Sohee Oh, So Yeon Kim, Young Mi Jung, Sun Min Kim, Chan-Wook Park, Jong Kwan Jun, Byoung Jae Kim, Joong Shin Park

**Affiliations:** 1 Department of Obstetrics and Gynecology, Seoul National University College of Medicine Seoul National University Hospital, 101 Daehak-ro, Jongno-gu, Seoul, 03080 South Korea; 2 Department of Biostatistics, Seoul Metropolitan Government Seoul National University Boramae Medical Center, Seoul, South Korea; 3 Department of Obstetrics and Gynecology, Seoul Metropolitan Government Seoul National University Boramae Medical Center, Seoul, South Korea

**Keywords:** High-throughput screening, Genetics research, Chromosome abnormality

## Abstract

To examine the detection performance of a peptide nucleic acid (PNA) probe-based real-time time polymerase chain reaction (PCR) assay to detect common aneuploidies. Using amniotic fluid samples, PNA probe based real-time PCR (Patio DEP Detection Kit; SeaSun Biomaterials, Korea) assay was performed. PNA probe was designed to hybridize to similar sequences located on different segments of target chromosomes (21, 18, and 13) and a reference chromosome. Amplification of target sequences and melting curve analysis was performed. When analyzing the melting curve, the ratio of the peak height of the target and reference chromosome was calculated and determined as aneuploidy if the ratio of peak height was abnormal. All the results from the PNA probe-based real-time PCR and melting curve analyses were compared to those from conventional karyotyping. Forty-two cases with common aneuploidies (24 of trisomy 21, 12 of trisomy 18, and 6 of trisomy 13) and 131 cases with normal karyotype were analyzed. When comparing the karyotyping results, the sensitivity and specificity of the PNA probe-based real-time PCR assay were both 100%. The level of agreement was almost perfect (k = 1.00). PNA real-time PCR assay is a rapid and easy method for detecting common aneuploidies.

## Introduction

In recent decades, many diagnostic methods for detecting fetal aneuploidies have been developed. Karyotyping is a standard diagnostic method for detecting fetal aneuploidy. However, karyotyping takes at least 2 weeks to yield results due to the requirement for culturing cell. To overcome this limitation, rapid molecular methods such as fluorescence in situ hybridization (FISH), quantitative fluorescence polymerase chain reaction (QF-PCR) and multiplex ligation-dependent probe amplification (MLPA) have been developed^1,2^.

FISH makes use of fluorescently labelled sequences and is used to detect common aneuploidies under the microscope^3^. It does not require deoxyribonucleic acid (DNA) extraction and cell culture, however, it requires a larger sample volume and it is labor-intensive and expensive test^1^. QF-PCR and MLPA are based on polymerase chain reaction (PCR) amplification of DNA short tandem repeat (STR) marker and of ligated probe, respectively^4,5^. They require additional analysis after PCR using a genetic analyzer to determine sequence copy number.

New strategies using real-time PCR, which does not require analysis using a genetic analyser after the PCR step, have been introduced^6–10^. These methods reduce test time because additional DNA sequencing is not required. Furthermore, multiple single nucleotide polymorphisms (SNPs) can be analyzed concurrently using multiple fluorescently labelled probes, enabling tests for several types of aneuploidy simultaneously^10^.

Recently, peptide nucleic acid (PNA) probe-based real-time PCR, a simple and rapid detection test for fetal chromosomal abnormalities, was developed. PNA probes are dual-labelled, random-coiled, self-quenching probes^11^. The probes comprise a short target-specific sequence, with a fluorophore and a dabsyl moiety attached at either end. In contrast to other types of molecular probes, PNA probes are not degraded by DNA polymerase during PCR elongation; thus, after PCR amplification, PNA probes can selectively form stable duplexes with the target. Thus, PNA probes have high binding affinity to complementary nucleic acids, and biological stability due to their uncharged nature and peptide bond-linked backbone^12,13^. PNA probes generate a large difference in melting temperature (ΔTm) between perfect match and single mismatch. As a result of these advantages, PNA probes have been widely applied in molecular biology^14,15^.

The objective of this study was to assess test performances of this new method, PNA probe-based real-time PCR, for the detection of aneuploidy using amniotic fluid.

## Methods

### Settings

Amniotic fluid samples were collected from women who underwent amniocentesis for clinical indication at Seoul National University Hospital and Seoul Metropolitan Government Seoul National University Boramae Medical Center in South Korea. We performed PNA-probe based real-time PCR and melting curve analysis and compared the results with those obtained from conventional karyotyping. This study was approved by the Institutional Review Board of Seoul National University Hospital (approval No. 1704-026-843) and Seoul Metropolitan Government Seoul National University Boramae Medical Center (approval No. 30-2017-7). All methods were carried out in accordance with the Declaration of Helsinki. Informed consent was obtained from all subjects for the collection and use of AF samples for research purposes.

### Method of karyotyping

The amniotic fluid was centrifuged at 182*g* (1000 rpm) for 7 min. Cell pellet was resuspended after adding 3 ml of AmnoMax medium (Gibco, NY, USA). Cells were dispended in each culture dish and monitored for 10–14 days at 37 °C with 5% CO_2_. When the cells become sufficient, 80 μl (10 μg/ml) of colcemid solution (Gibco, NY, USA) was added to arrest cell division in the metaphase. It was incubated for 40 min at 37 °C, and then treated with hypotonic solution (0.0375 M KCL) for 20 min. Then, 500 μl of Carnoy’s solution (3:1 methanol/glacial acetic acid fixative) was added to the edge for the coverslip. The supernatants were removed, and 2 ml of Carnoy’s solution was added, this step was repeated two times. Slide was made and stained with GTG banding (G-bands by trypsin using Giemsa). At least 25 metaphases from two independent culture dishes were observed under microscope. Karyotype was analyzed using ChIPS-Karyo (GenDix, Seoul, Korea) (Supplementary Fig. 1). The nomenclature of chromosome results followed as International System for Human Cytogenetic Nomenclature (2016).

### The real-time PCR based diagnostic kit for the detection of common trisomies

**Figure 1:**
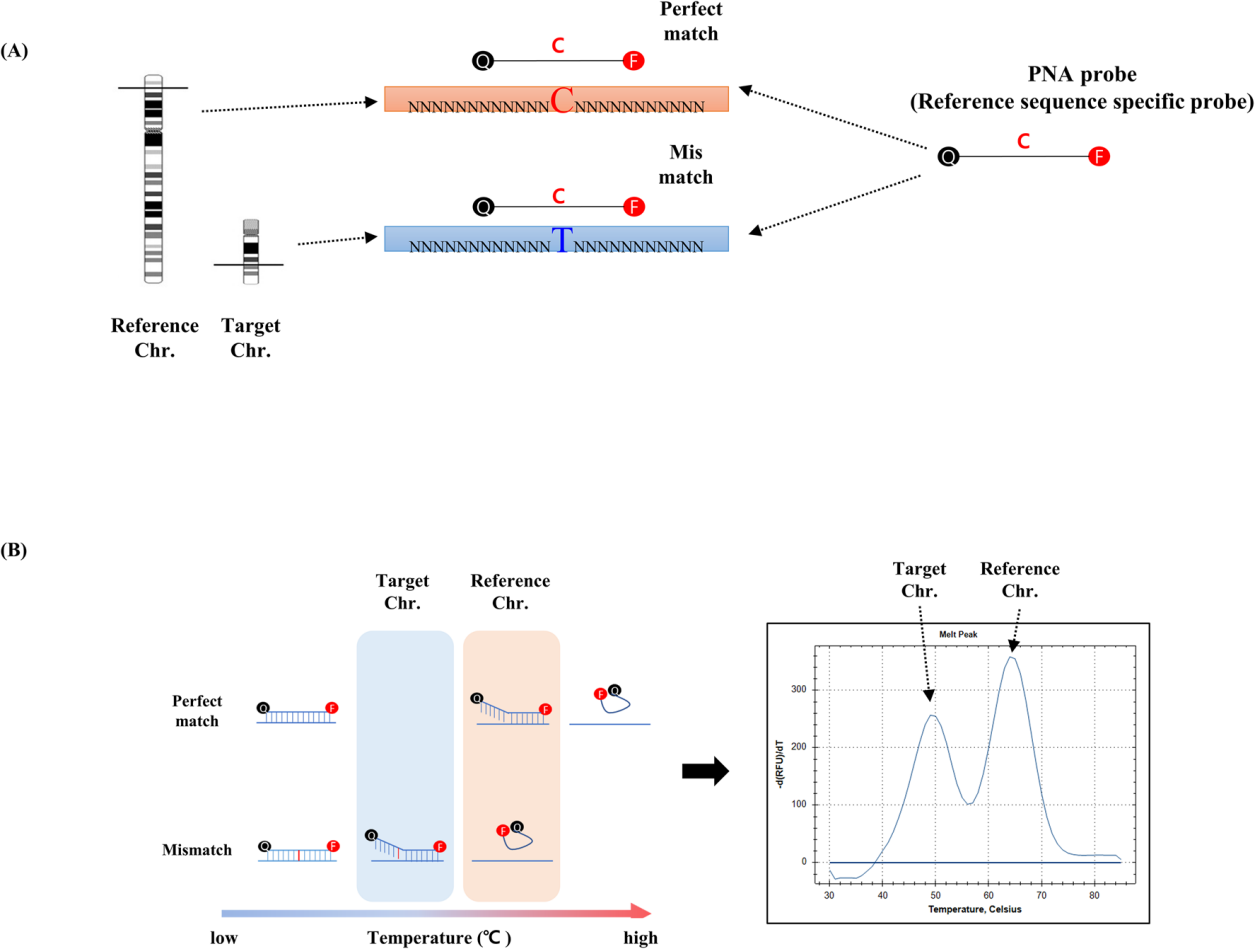
The principles of PNA probe-based real-time PCR combined with melting curve analysis. (**A**) A PNA probe was designed to detect the single nucleotide differences between target chromosome (21, 18, 13) and the reference chromosome. (**B**) Melting curve analyses were of interest due to melting point differences between perfect match and mismatch.

Patio DEP Detection Kit (SeaSun Biomaterials, Daejeon, Korea) consists of 2× PCR premix, as well as the primer and PNA probe mixture (DEP set 1, 2, 3, 4). The PNA probe was designed to hybridise to paralogs of target and reference chromosomes which have the same sequence but for a single nucleotide difference. This resulted in a ΔTm between perfect match and single mismatch. (Fig. 1). Primer and probe sequences cannot be revealed due to the manufacturer’s copyright policy. Each PNA probe was fluorescently labelled with Fluorescein amidite (FAM) for trisomy 21, Texas-Red for trisomy 18, and Hexachloro-fluorescein (HEX) for trisomy 13. The Patio DEP Detection Kit consists of four sets which include three markers for aneuploidy 21, 18, and 13 in each set. The limit of detection of the kit was 15 ng of DNA.

### Real-time PCR and melting curve analysis

Genomic DNA (gDNA) was isolated from amniotic fluid samples using QIAamp DNA Mini Kit (QIAGEN) according to the manufacturer’s instructions. Using isolated DNA from amniotic fluid (AF), real-time PCR was performed using the Patio DEP Detection Kit (SeaSun Biomaterials, Daejeon, Korea) with a CFX96 Real-Time PCR Detection system (Bio-Rad, Hercules, CA, USA).

Real-time PCR was performed in a final volume of 20-μl containing 10 μl of 2× qPCR PreMix, 7 μl of primer and PNA probe mixture (Patio DEP Detection Kit, DEP set 1, 2, 3, 4), and 3 μl of DNA template (5 ng/μl). Each mixture was applied to individual well and four wells were necessary for testing one case. The reaction conditions for amplification were 95 °C for 10 min; 45 cycles of 95 °C for 30 s, 58 °C for 45 s, and 72 °C for 45 s; followed by melting point analysis.

Melting point analysis was performed using a denaturation step of 95 °C for 5 min; 1 min hybridisation steps of 75 °C, 55 °C, and 45 °C; and a stepwise temperature increase from 30 to 90 °C in 1 °C intervals, with a 5 s interval between each step. The experiments for optimal temperature increase were described in “Supplemental information”. The data were analysed using Bio-Rad CFX manager v1.6 software (Bio-Rad).

### Interpretation of results

**Figure 2:**
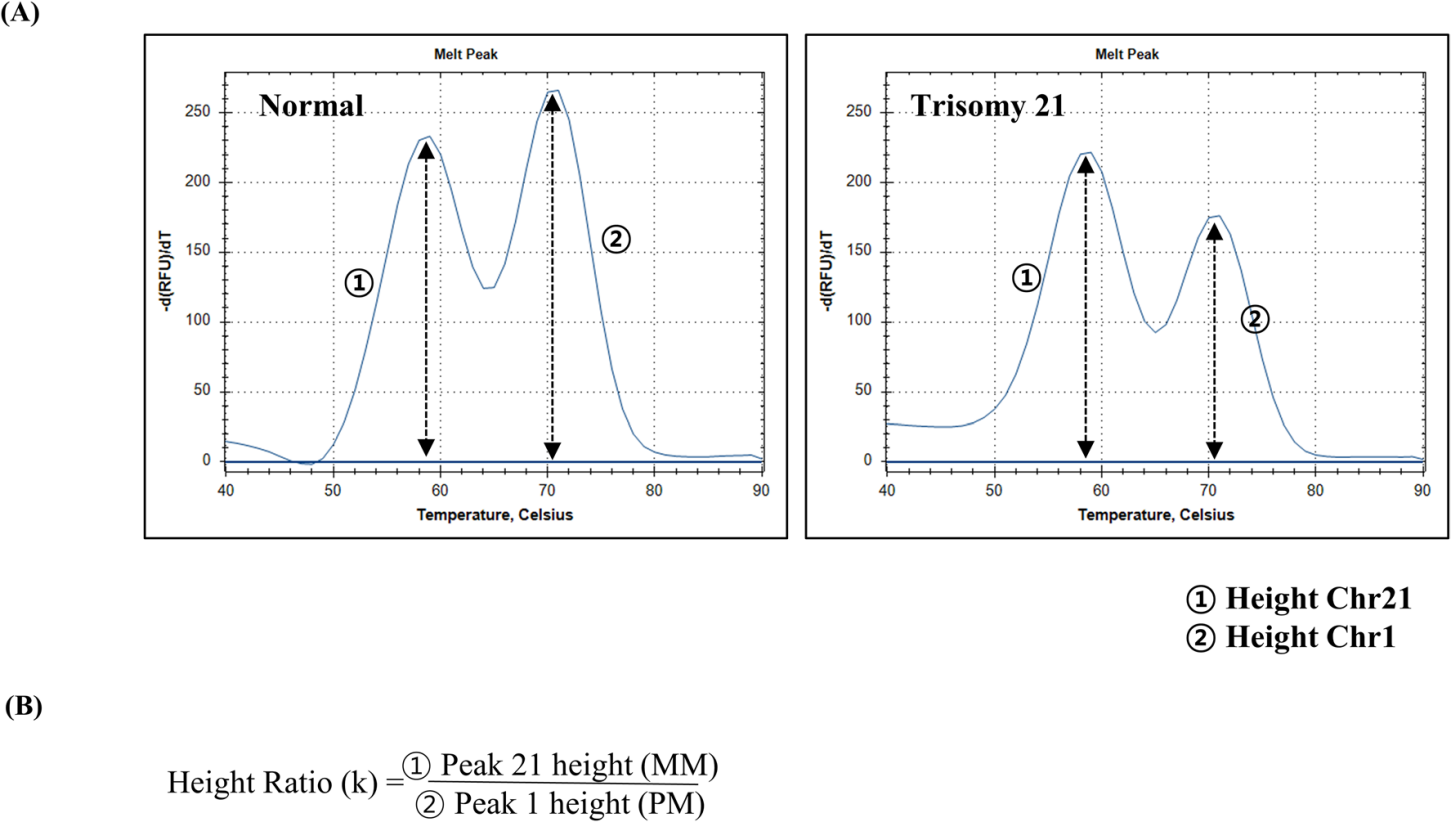
Examples of melting curve analysis for trisomy 21. (**A**) Melting curve patterns for normal versus trisomy 21. The low temperature (mismatch temperature) peaks indicate the quantification of chromosome 21 and the high temperature (perfect match temperature) peaks indicate quantification of the reference chromosome. (**B**) Calculation formula of the peak height ratio (K).

After amplification and melting curve analysis, the peak heights under melting curves from perfect match temperature (PMT) and mismatch temperature (MMT) which indicate the quantification of chromosomes was calculated (Fig. 2). Then, the peak height ratio (K) was calculated by comparing peak height of PMT to peak height of MMT. Trisomy has one more chromosome than normal, and hence trisomy has an abnormal peak height ratio. The abnormal ration is distinguished from a normal ratio by analysing the peak height ratio of the target and reference chromosomes. An abnormal K value was determined according to the cut-off K value provided by the kit manufacturer (Supplementary Table S1). If an abnormal ratio (K) was identified in three or more of the four sets on the same chromosome, it was judged as a trisomy of the chromosome.

### Statistical analysis

For evaluating diagnostic performance, sensitivity and specificity, and positive and negative predictive values with a 95% confidence interval, the Wilson interval method was used^16^. A kappa (*k*) index was calculated to determine the agreement of the two diagnostic methods for aneuploidy. IBM SPSS Statistics version 23.0 software (IBM Inc., Armonk, NY) was used for the analyses.

## Results

### Study population

A total of 173 amniotic fluid samples were used in the analysis, including 42 cases with common aneuploidies (24 with trisomy 21, 12 with trisomy 18, and 6 with trisomy 13) and 131 normal control cases.

### Detection performance of PNA based real-time PCR

All cases were successfully analyzed by PNA probe-based real-time PCR. In Table 1. we summaries the detection performance of PNA probe-based real-time PCR compared with conventional karyotyping, when each trisomy was judged when the abnormal height ratio (K) was identified in three or more of the four sets. All of the results from the PNA based real-time PCR assay were consistent with the results obtained from karyotyping. The sensitivity and specificity of the PNA based real-time PCR assay were both 100% (sensitivity: 100% (91.6–100%), specificity: 100% (97.2–100%)). The *k* index was 1.0 and these two methods were in perfect agreement.

**Table 1:** A comparison between PNA probe-based real-time PCR and conventional karyotyping for the detection of fetal aneuploidies.

a) Results of PNA probe-based real-time PCR for detecting each aneuploidy					
Results for aneuploidy	Karyotyping				Total
	Normal	Trisomy21	Trisomy 18	Trisomy 13	
PNA probe based real-time PCR					
Normal	131	0	0	0	131
Trisomy 21	0	24	0	0	24
Trisomy 18	0	0	12	0	12
Trisomy 13	0	0	0	6	6
Total	131	24	12	6	173

b) Diagnostic performance of PNA probe-based real-time PCR					
	Sensitivity	Specificity	PPV	NPV	
Overall (%)	100 (91.6–100)	100 (97.2–100)	100 (91.6–100)	100 (97.2–100)	

Data are presented as % (95% confidence interval).

*PNA* peptide nucleic acid, *PCR* polymerase chain reaction, *PPV* positive predictive value, *NPV* negative predictive value.

### Representative melting curve patterns and the distribution of k-values

**Figure 3:**
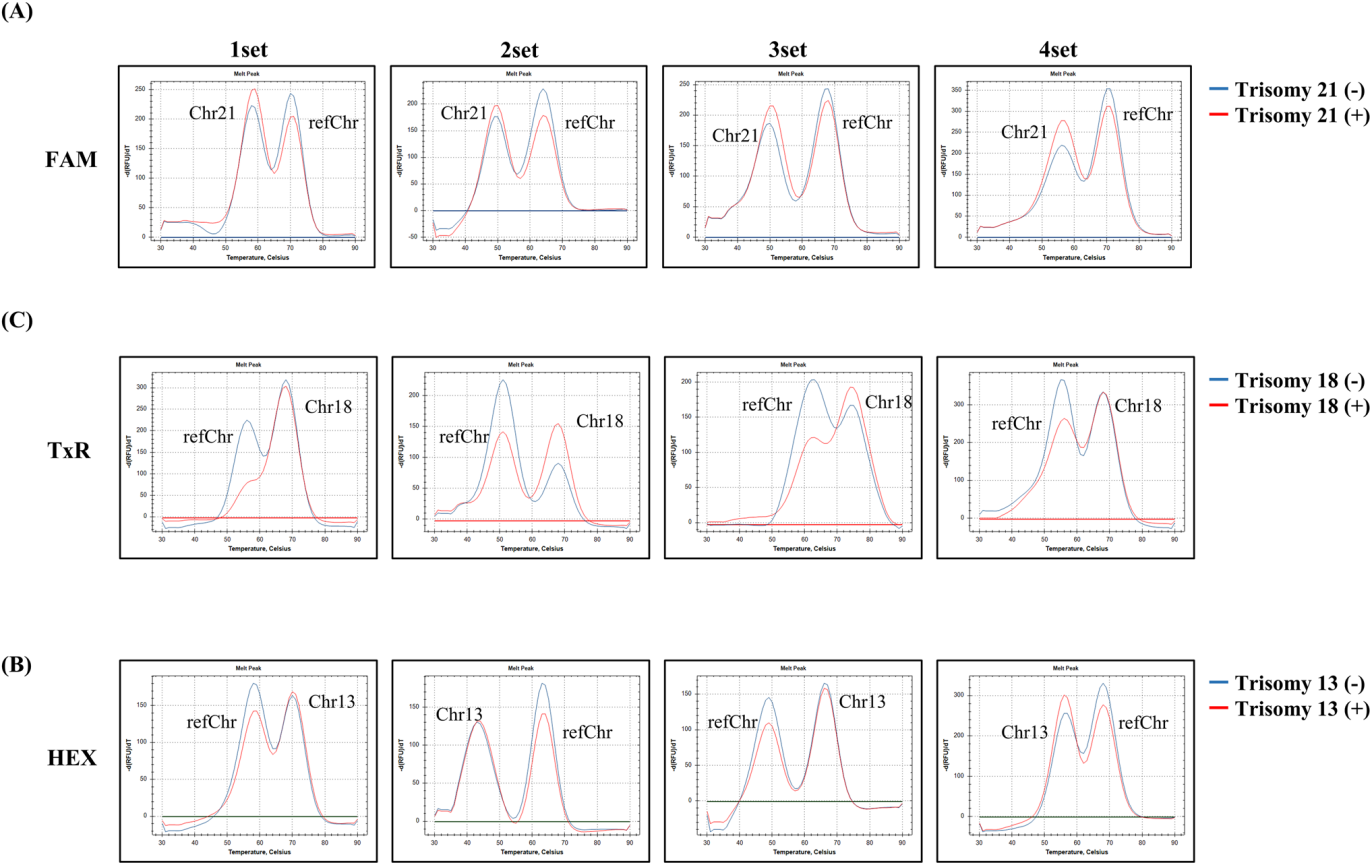
Representative melting curve patterns for trisomy 13, 18 and 21 compared with a normal melting curve. In the case of trisomy 13, high temperature peaks of set 1 and 3 indicate the quantification of chromosome 13, otherwise low temperature peaks of set 2 and 4 indicate the quantification of Chr13. In the case of trisomy 18, all of the high temperature peaks indicate the quantification of chromosome 18. In the case of trisomy 21, all of the low temperature peaks indicate the quantification of chromosome 21. In the case of trisomy 21 (+) (red line) in the FAM channel, the patterns of the low temperature peak (chromosome 21) and the high temperature peak (reference Chr.) were different from the trisomy 21 (−) in all four sets. The ratios of melting peak height were always higher in trisomy 21 (+) than trisomy 21 (−). In the same manner, the ratios of melting peak height were lower in trisomy 18 (+) than trisomy 18 (−) in all sets and the patterns of the melting curve were different between trisomy 18 (+) and trisomy 18 (−). The melting curve patterns of trisomy 13 (+) were also different from trisomy 13 (−).

**Figure 4:**
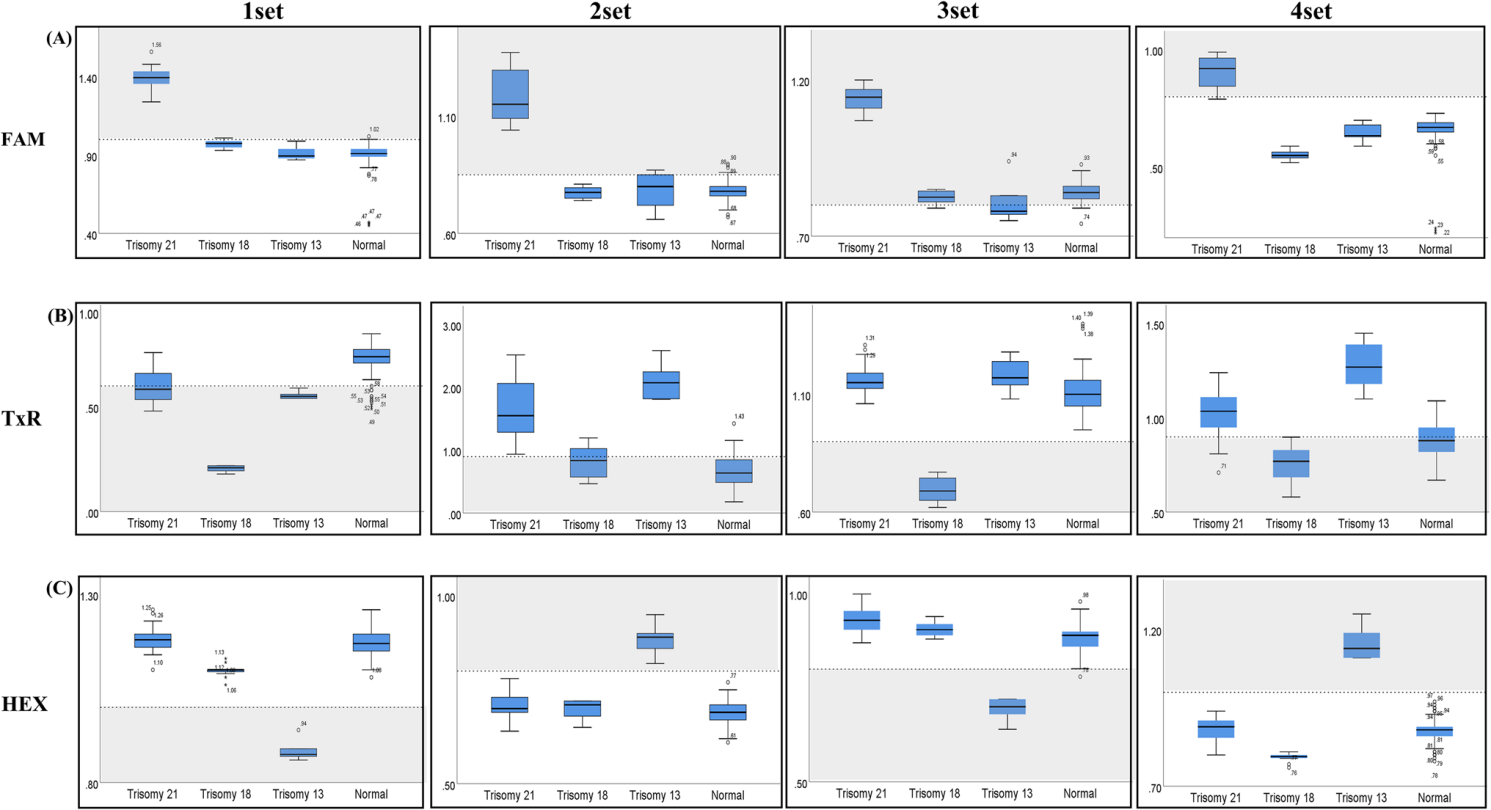
The distribution of K-values of all samples. Gray zone means abnormal K-value section. In the FAM channel, K-values of trisomy 21 were included in the gray zone in all sets of the Patio DEP Detection Kit, whereas those of other trisomies and normal cases fell out of the gray zone. Similarly, K-values of trisomy 13 in the HEX channel and those of trisomy 18 in TxR channel were included in the gray zone, and they were distinguished from the K-value distribution of other trisomies and normal control.

Figure 3 demonstrates representative melting curve patterns for trisomy 21, 18 and 13, respectively. The aneuploidy is determined by checking peak height ratio (K) calculated from the peak height of the target and reference chromosomes. Figure 4 shows the distribution of k-values of all samples. Most of the probes showed good discrimination. The cut-off values of K, for each marker, are shown in the Supplementary Table S1. The Actual k-values of all samples are presented in the Supplementary Table S2.

## Discussion

The principal finding of the current study is that: (1) the accuracy of PNA probe-based real time-PCR and melting curve analyses to identify aneuploidies of chromosomes 21, 18, and 13 was 100% sensitivity and specificity; (2) compared with karyotyping, the PNA probe-based real time-PCR method had a shorter test time with equally accurate results (Table 1).

Several studies have been conducted to identify aneuploidies using real time PCR without a post PCR step^6–10^. In an early pilot study using real-time PCR, normal and trisomy 21 were distinguished by the difference in threshold cycle value^6^. In another study wherein the authors conducted melting curve analysis of multiple SNPs using real-time PCR, identification of trisomy 21 was 90% accurate^7^. However, this assay was complex due to the need to select the most informative SNPs and difficulties were experienced due to lack of reproducibility arising from heterozygosity of SNPs. In addition, the assay could only be used to identify trisomy 21. For identifying other common trisomies, as well as trisomy 21, several studies have been conducted such as MLPA with real-time PCR and high resolution melting curve analysis^8,9^, MLPA with real time PCR has the advantage of combining the accuracy of MLPA and the simplicity of real-time PCR. However, this method has a complex and time consuming (approximately 20 h) data analysis process. Although high resolution melting curve analysis was also shown to be an accurate method for the determination of common trisomies, this method could not distinguish aneuploidies simultaneously in one tube.

According to a recent analysis of aneuploidies using melting curve analysis by quadruplex real-time PCR, the assay could be used to identify trisomies simultaneously with 100% accuracy when analyzing 131 samples^10^. This method used segmental duplication with single nucleotide differences located on the target and reference chromosomes and relied upon the calculation of a peak height ratio. It has similar methodology to that of the current study and the result is consistent with our findings.

To our knowledge, our study is the first study to evaluate the use of PNA probes for the identification of aneuploidy. PNA probes have favorable hybridization properties as they are uncharged and have a peptide bond-linked back bone^13^. Due to these characteristics, PNA probes can be designed to be shorter than DNA probes hence enabling a greater ΔTm mismatch between wild type and mutant^11^. In addition, the PNA probe supplied with the kit was designed from four segmental duplications of each trisomy, and this enhanced the ability to differentiate between the SNPs. As a result of this ability, the PNA based real time PCR assay was used to identify common trisomies with 100% sensitivity and specificity.

When using a 96-well plate, the results from 24 patients were obtained within 3 h through a single step assay. The automated manner of this method can provide physicians with results more easily than other more labor-intensive methods, and can do so in a timely manner.

We examined AF from two centers, and additional multi-center studies with larger study populations are required to verify the results of the current study. In addition, a cost effectiveness analysis should be performed prior to application in clinical practice. Although this method reduces the psychological anxiety of the mother by providing rapid results, this is not a diagnostic test but a supplemental method. This assay was validated from samples with non-mosaic trisomy 21, 18, and 13, and hence, further studies are required to verify the assay performance for the detection of mosaicisms. It is also necessary to develop the test beyond chromosomes 21, 18, and 13, so as to detect other types of aneuploidy.

In conclusion, PNA probe-based real-time PCR assay is a rapid and easy method for detecting common aneuploidies. The automated manner of this method and the characteristic which does not require analysis using a genetic analyser after the PCR step can provide physicians with results more easily than other more labour-intensive methods.

## Supplementary Information

Supplementary Figure 1.

Supplementary Information.

Supplementary Tables.

## Data Availability

The datasets used and analysed during the current study are available from the corresponding author on reasonable request.
