# Supplementary figures and images for: Simple and rapid detection of common fetal aneuploidies using peptide nucleic acid probe-based real-time polymerase chain reaction
Source: Sci Rep. 2022 Jan 7;12:150. doi: 10.1038/s41598-021-02507-5 (PMC8742004; doi:10.1038/s41598-021-02507-5)

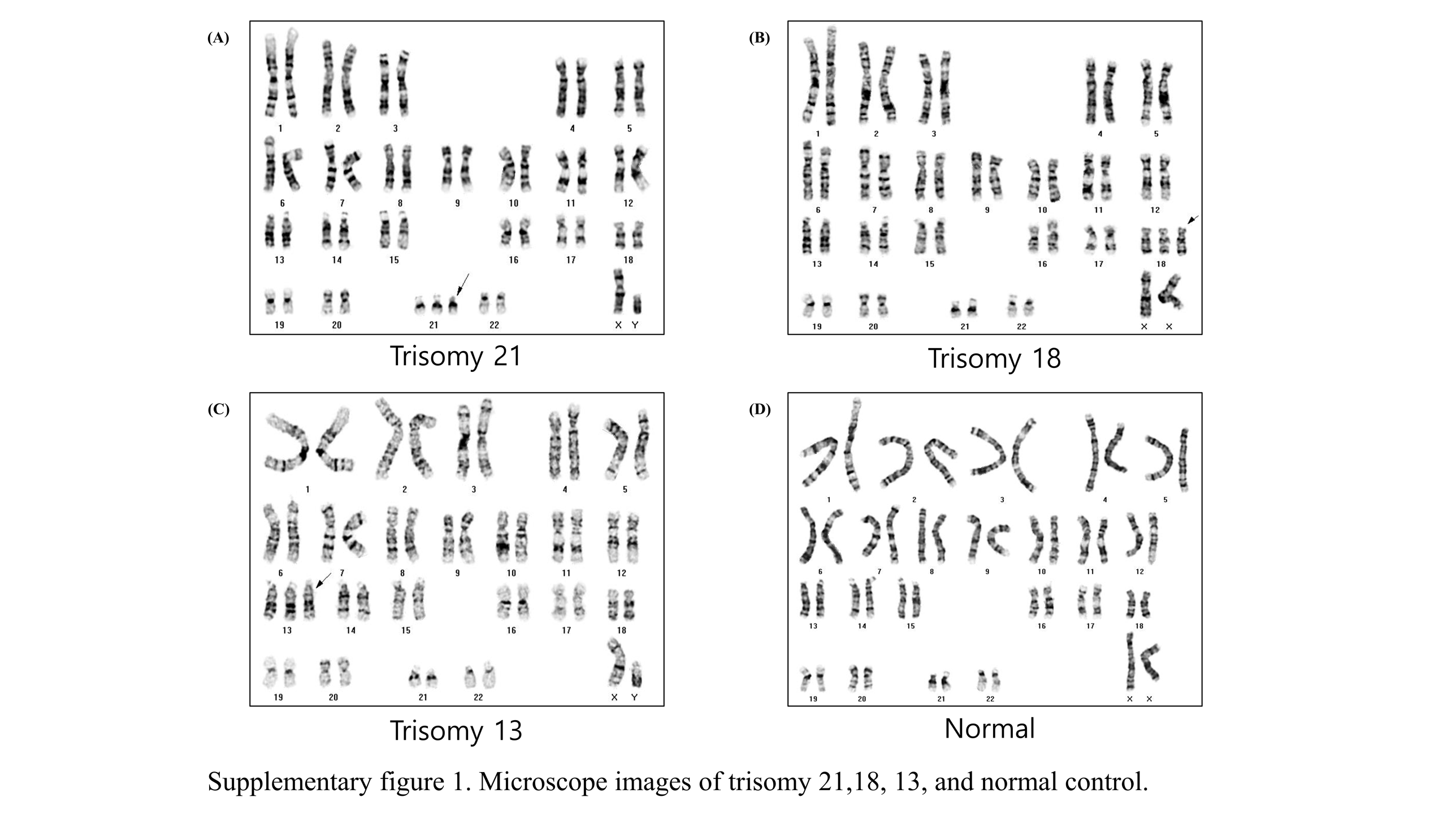

Supplement: Supplementary file 1 — Supplementary Figure 1. [file 41598_2021_2507_MOESM1_ESM.tif]
