# Supplementary material for: Simple and rapid detection of common fetal aneuploidies using peptide nucleic acid probe-based real-time polymerase chain reaction
Source: Sci Rep. 2022 Jan 7;12:150. doi: 10.1038/s41598-021-02507-5 (PMC8742004; doi:10.1038/s41598-021-02507-5)
Supplement: Supplementary file 2 — Supplementary Information. [file 41598_2021_2507_MOESM2_ESM.docx]

- Supplementary methods -

**- The melting experiments**

In this study, melting point analysis was performed using a denaturation step of 95°C for 5 min; 1 min hybridization steps of 75°C, 55°C, and 45°C; and a stepwise temperature increase from 30 to 90°C in 1°C intervals, with a 5 s interval between each step. The data were analyzed using Bio-Rad CFX manager v1.6 software (Bio-Rad).

To optimize this step, several conditions of MA were tested with Patio DEP Detection Kit. Melting curves of PCR amplicons were tested with temperatures ranging from 30°C to 90°C with a 0.5°C and 1 °C increase in temperature every 5 and 10 seconds using normal genomic DNA (Hela genomic DNA, NEB).

>Results


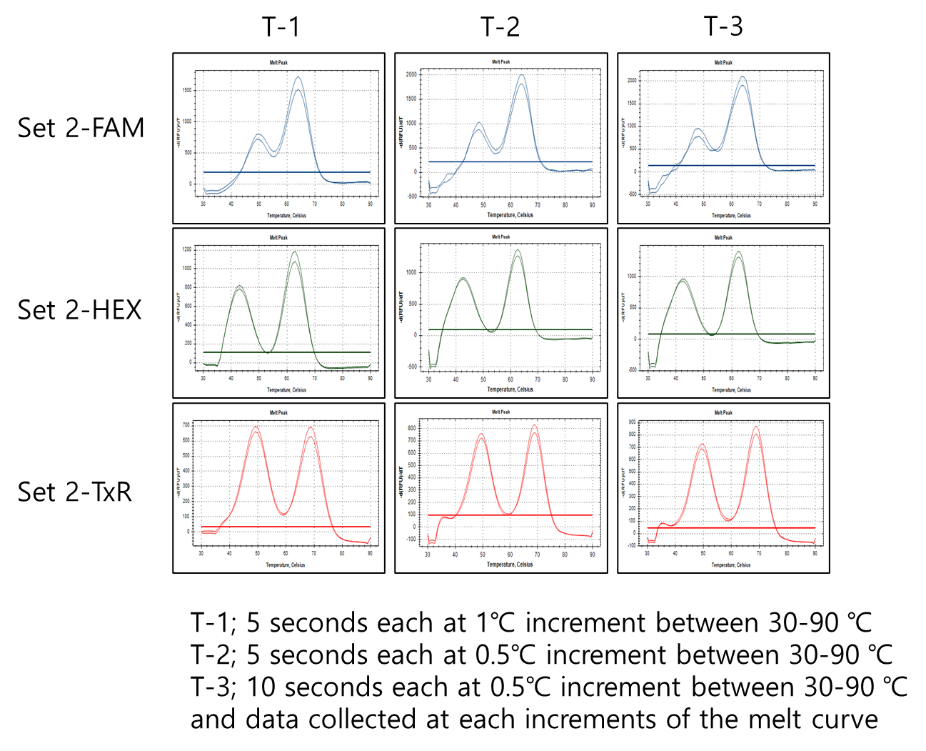


Melting curves ranging from 30°C to 90°C, increasing 0.5°C per cycle and measured fluorescence for 5 and 10 seconds at each PCR cycle were took long Melting analysis PCR time and shapes of melting curves were not sharp.

However, melting curves increased 1°C per cycle and measured fluorescence for 5 seconds at each PCR cycle were took shorter time than other MA conditions tested. It took about 30 minutes and other conditions described above took about 55 and 70 minutes for each. Also, shape of melting curves was sharper and has more accurate melting temperatures in FAM channel. K-values of all fluorescence channel (FAM, HEX and Texas Red) were similar in melt curve steps tested.
