# Supplementary material for: Simple and rapid detection of common fetal aneuploidies using peptide nucleic acid probe-based real-time polymerase chain reaction
Source: Sci Rep. 2022 Jan 7;12:150. doi: 10.1038/s41598-021-02507-5 (PMC8742004; doi:10.1038/s41598-021-02507-5)
Supplement: Supplementary file 3 — Supplementary Tables. [file 41598_2021_2507_MOESM3_ESM.docx]

Supplementary Table S1. Cut-off value of K for determining trisomy.

| **Tube** | **Fluorescents** | **Gene** | **PM** | **MM** | **K=MM/PM** | |
| --- | --- | --- | --- | --- | --- | --- |
|  |  |  |  |  | **Normal** | **Trisomy** |
| **Set 1** | FAM | DS-1 | 69℃ | 58℃ | K < 1.0 | K ≥ 1.0 |
|  | Texas Red | ES-1 | 68℃ | 54℃ | K > 0.6 | K ≤ 0.6 |
|  | HEX | PS-1 | 70℃ | 58℃ | K > 1.0 | K ≤ 1.0 |
| **Set 2** | FAM | DS-2 | 64℃ | 50℃ | K < 0.9 | K ≥ 0.9 |
|  | Texas Red | ES-2 | 69℃ | 51℃ | K > 0.9 | K ≤ 0.9 |
|  | HEX | PS-2 | 64℃ | 44℃ | K < 0.8 | K ≥ 0.8 |
| **Set 3** | FAM | DS-3 | 66℃ | 50℃ | K < 0.8 | K ≥ 0.8 |
|  | Texas Red | ES-3 | 77℃ | 65℃ | K > 0.9 | K ≤ 0.9 |
|  | HEX | PS-3 | 67℃ | 49℃ | K > 0.8 | K ≤ 0.8 |
| **Set 4** | FAM | DS-4 | 70℃ | 58℃ | K < 0.8 | K ≥ 0.8 |
|  | Texas Red | ES-4 | 68℃ | 55℃ | K > 0.9 | K ≤ 0.9 |
|  | HEX | PS-4 | 68℃ | 56℃ | K < 1.0 | K ≥ 1.0 |

Supplementary Table S2. K-values of all samples

|  | **set1** | | | **set2** | | | **set3** | | | **set4** | | | **Results** |
| --- | --- | --- | --- | --- | --- | --- | --- | --- | --- | --- | --- | --- | --- |
|  | **FAM** | **Texas Red** | **HEX** | **FAM** | **Texas Red** | **HEX** | **FAM** | **Texas Red** | **HEX** | **FAM** | **Texas Red** | **HEX** |  |
| 1 | 1.25 | 0.56 | 1.15 | 1.30 | 1.88 | 0.77 | 1.07 | 1.13 | 0.87 | 0.84 | 1.02 | 0.84 | Down |
| 2 | 1.40 | 0.54 | 1.10 | 1.29 | 2.52 | 0.78 | 1.10 | 1.10 | 0.91 | 0.85 | 1.17 | 0.85 | Down |
| 3 | 1.24 | 0.48 | 1.17 | 1.29 | 2.34 | 0.75 | 1.13 | 1.13 | 0.93 | 0.84 | 1.02 | 0.80 | Down |
| 4 | 1.37 | 0.54 | 1.23 | 1.37 | 2.02 | 0.69 | 1.18 | 1.16 | 0.98 | 0.82 | 0.97 | 0.87 | Down |
| 5 | 1.39 | 0.57 | 1.21 | 1.33 | 1.80 | 0.71 | 1.19 | 1.16 | 0.97 | 0.87 | 0.94 | 0.86 | Down |
| 6 | 1.40 | 0.57 | 1.20 | 1.28 | 1.44 | 0.68 | 1.15 | 1.17 | 0.93 | 0.88 | 0.81 | 0.91 | Down |
| 7 | 1.33 | 0.55 | 1.18 | 1.32 | 1.20 | 0.69 | 1.08 | 1.26 | 0.88 | 0.89 | 0.71 | 0.86 | Down |
| 8 | 1.40 | 0.52 | 1.19 | 1.27 | 1.30 | 0.70 | 1.08 | 1.31 | 0.95 | 0.89 | 0.82 | 0.89 | Down |
| 9 | 1.36 | 0.49 | 1.16 | 1.31 | 1.65 | 0.69 | 1.10 | 1.27 | 0.91 | 0.84 | 0.87 | 0.85 | Down |
| 10 | 1.37 | 0.52 | 1.16 | 1.18 | 2.28 | 0.71 | 1.13 | 1.29 | 0.94 | 0.82 | 0.96 | 0.85 | Down |
| 11 | 1.31 | 0.53 | 1.18 | 1.37 | 2.14 | 0.73 | 1.14 | 1.20 | 0.91 | 0.79 | 0.94 | 0.83 | Down |
| 12 | 1.42 | 0.52 | 1.18 | 1.04 | 2.11 | 0.70 | 1.16 | 1.15 | 0.91 | 0.93 | 1.14 | 0.90 | Down |
| 13 | 1.56 | 0.60 | 1.21 | 1.05 | 1.90 | 0.70 | 1.18 | 1.06 | 0.89 | 0.91 | 1.04 | 0.86 | Down |
| 14 | 1.42 | 0.69 | 1.15 | 1.11 | 1.57 | 0.69 | 1.19 | 1.14 | 1.00 | 0.97 | 1.08 | 0.92 | Down |
| 15 | 1.38 | 0.71 | 1.16 | 1.10 | 1.21 | 0.73 | 1.16 | 1.11 | 0.98 | 0.96 | 1.05 | 0.93 | Down |
| 16 | 1.35 | 0.65 | 1.14 | 1.06 | 1.06 | 0.76 | 1.12 | 1.18 | 0.98 | 0.96 | 1.03 | 0.92 | Down |
| 17 | 1.48 | 0.69 | 1.19 | 1.07 | 1.24 | 0.69 | 1.12 | 1.09 | 0.96 | 0.97 | 1.06 | 0.94 | Down |
| 18 | 1.37 | 0.72 | 1.19 | 1.12 | 1.30 | 0.70 | 1.09 | 1.17 | 0.89 | 0.94 | 1.16 | 0.91 | Down |
| 19 | 1.44 | 0.62 | 1.19 | 1.05 | 1.52 | 0.70 | 1.15 | 1.15 | 0.89 | 0.99 | 1.03 | 0.89 | Down |
| 20 | 1.44 | 0.63 | 1.15 | 1.12 | 1.51 | 0.71 | 1.19 | 1.20 | 0.90 | 0.95 | 1.17 | 0.90 | Down |
| 21 | 1.45 | 0.67 | 1.19 | 1.08 | 2.13 | 0.68 | 1.15 | 1.13 | 0.94 | 0.93 | 1.24 | 0.91 | Down |
| 22 | 1.31 | 0.76 | 1.25 | 1.16 | 1.53 | 0.72 | 1.15 | 1.15 | 0.91 | 0.98 | 1.19 | 0.91 | Down |
| 23 | 1.45 | 0.63 | 1.18 | 1.14 | 0.94 | 0.64 | 1.14 | 1.08 | 0.94 | 0.97 | 1.06 | 0.92 | Down |
| 24 | 1.43 | 0.63 | 1.26 | 1.11 | 1.28 | 0.75 | 1.20 | 1.12 | 0.95 | 0.97 | 1.04 | 0.89 | Down |
| 25 | 0.97 | 0.22 | 1.10 | 0.81 | 0.50 | 0.72 | 0.81 | 0.70 | 0.90 | 0.59 | 0.70 | 0.79 | Edward |
| 26 | 0.95 | 0.22 | 1.10 | 0.75 | 0.79 | 0.68 | 0.82 | 0.64 | 0.92 | 0.55 | 0.84 | 0.80 | Edward |
| 27 | 0.93 | 0.21 | 1.10 | 0.75 | 1.20 | 0.72 | 0.81 | 0.62 | 0.88 | 0.53 | 0.899 | 0.80 | Edward |
| 28 | 0.98 | 0.18 | 1.10 | 0.77 | 1.04 | 0.72 | 0.79 | 0.67 | 0.89 | 0.55 | 0.83 | 0.77 | Edward |
| 29 | 1.01 | 0.19 | 1.12 | 0.76 | 0.96 | 0.72 | 0.85 | 0.68 | 0.91 | 0.54 | 0.77 | 0.79 | Edward |
| 30 | 0.95 | 0.19 | 1.13 | 0.80 | 0.89 | 0.71 | 0.85 | 0.65 | 0.92 | 0.57 | 0.72 | 0.80 | Edward |
| 31 | 0.98 | 0.20 | 1.06 | 0.78 | 0.63 | 0.66 | 0.83 | 0.65 | 0.92 | 0.55 | 0.65 | 0.76 | Edward |
| 32 | 0.96 | 0.20 | 1.10 | 0.78 | 0.52 | 0.71 | 0.85 | 0.72 | 0.94 | 0.58 | 0.58 | 0.79 | Edward |
| 33 | 0.94 | 0.22 | 1.10 | 0.80 | 0.47 | 0.65 | 0.82 | 0.75 | 0.88 | 0.54 | 0.67 | 0.81 | Edward |
| 34 | 0.99 | 0.22 | 1.08 | 0.79 | 0.79 | 0.68 | 0.84 | 0.75 | 0.89 | 0.56 | 0.80 | 0.81 | Edward |
| 35 | 1.01 | 0.21 | 1.10 | 0.75 | 1.08 | 0.69 | 0.81 | 0.74 | 0.89 | 0.55 | 0.83 | 0.79 | Edward |
| 36 | 0.99 | 0.21 | 1.09 | 0.74 | 1.02 | 0.71 | 0.83 | 0.77 | 0.92 | 0.52 | 0.77 | 0.80 | Edward |
| 37 | 0.99 | 0.59 | 0.94 | 0.87 | 2.25 | 0.89 | 0.78 | 1.24 | 0.68 | 0.70 | 1.45 | 1.25 | Patau |
| 38 | 0.91 | 0.54 | 0.86 | 0.72 | 1.82 | 0.86 | 0.78 | 1.17 | 0.70 | 0.68 | 1.39 | 1.19 | Patau |
| 39 | 0.94 | 0.54 | 0.87 | 0.66 | 2.11 | 0.89 | 0.75 | 1.17 | 0.64 | 0.59 | 1.18 | 1.11 | Patau |
| 40 | 0.88 | 0.55 | 0.88 | 0.78 | 1.81 | 0.82 | 0.94 | 1.08 | 0.72 | 0.63 | 1.20 | 1.14 | Patau |
| 41 | 0.87 | 0.56 | 0.89 | 0.85 | 2.04 | 0.95 | 0.83 | 1.28 | 0.72 | 0.63 | 1.10 | 1.14 | Patau |
| 42 | 0.88 | 0.55 | 0.87 | 0.82 | 2.59 | 0.90 | 0.77 | 1.14 | 0.70 | 0.64 | 1.34 | 1.11 | Patau |
| 43 | 0.94 | 0.50 | 1.23 | 0.74 | 0.68 | 0.68 | 0.74 | 1.17 | 0.98 | 0.66 | 0.95 | 0.83 | Normal |
| 44 | 0.96 | 0.54 | 1.21 | 0.71 | 0.82 | 0.66 | 0.86 | 0.98 | 0.84 | 0.62 | 1.00 | 0.82 | Normal |
| 45 | 0.96 | 0.55 | 1.18 | 0.68 | 1.01 | 0.66 | 0.796 | 1.08 | 0.78 | 0.68 | 1.08 | 0.89 | Normal |
| 46 | 0.96 | 0.58 | 1.17 | 0.79 | 1.08 | 0.63 | 0.82 | 1.16 | 0.83 | 0.59 | 1.07 | 0.81 | Normal |
| 47 | 0.94 | 0.52 | 1.19 | 0.71 | 1.16 | 0.65 | 0.91 | 1.17 | 0.89 | 0.55 | 0.93 | 0.81 | Normal |
| 48 | 0.93 | 0.68 | 1.20 | 0.67 | 1.01 | 0.62 | 0.82 | 1.04 | 0.88 | 0.58 | 0.98 | 0.79 | Normal |
| 49 | 0.83 | 0.55 | 1.19 | 0.77 | 0.95 | 0.63 | 0.798 | 1.15 | 0.81 | 0.64 | 0.94 | 0.80 | Normal |
| 50 | 0.91 | 0.53 | 1.18 | 0.75 | 0.90 | 0.67 | 0.87 | 1.15 | 0.87 | 0.58 | 0.83 | 0.82 | Normal |
| 51 | 0.89 | 0.53 | 1.17 | 0.77 | 0.84 | 0.68 | 0.89 | 1.11 | 0.88 | 0.62 | 0.92 | 0.85 | Normal |
| 52 | 0.85 | 0.65 | 1.16 | 0.74 | 1.43 | 0.65 | 0.88 | 1.07 | 0.89 | 0.62 | 0.88 | 0.88 | Normal |
| 53 | 0.92 | 0.51 | 1.17 | 0.73 | 1.09 | 0.64 | 0.82 | 1.02 | 0.85 | 0.63 | 0.99 | 0.86 | Normal |
| 54 | 0.97 | 0.63 | 1.17 | 0.70 | 0.96 | 0.66 | 0.84 | 1.19 | 0.88 | 0.60 | 0.96 | 0.84 | Normal |
| 55 | 0.90 | 0.65 | 1.17 | 0.89 | 0.86 | 0.71 | 0.86 | 0.95 | 0.87 | 0.67 | 0.96 | 0.89 | Normal |
| 56 | 0.86 | 0.65 | 1.13 | 0.86 | 0.86 | 0.72 | 0.91 | 0.95 | 0.90 | 0.65 | 0.94 | 0.87 | Normal |
| 57 | 0.95 | 0.74 | 1.16 | 0.86 | 0.75 | 0.74 | 0.84 | 0.96 | 0.88 | 0.67 | 0.91 | 0.89 | Normal |
| 58 | 0.84 | 0.66 | 1.15 | 0.85 | 0.60 | 0.73 | 0.89 | 1.01 | 0.92 | 0.71 | 0.88 | 0.92 | Normal |
| 59 | 0.91 | 0.72 | 1.16 | 0.85 | 0.75 | 0.75 | 0.83 | 1.04 | 0.91 | 0.66 | 0.89 | 0.88 | Normal |
| 60 | 0.84 | 0.72 | 1.18 | 0.84 | 1.06 | 0.70 | 0.90 | 1.04 | 0.94 | 0.67 | 1.00 | 0.96 | Normal |
| 61 | 0.94 | 0.58 | 1.15 | 0.896 | 1.15 | 0.70 | 0.85 | 1.00 | 0.90 | 0.66 | 1.09 | 0.88 | Normal |
| 62 | 0.92 | 0.72 | 1.11 | 0.83 | 1.07 | 0.72 | 0.87 | 0.95 | 0.91 | 0.61 | 1.00 | 0.82 | Normal |
| 63 | 0.98 | 0.75 | 1.22 | 0.86 | 0.98 | 0.74 | 0.86 | 1.04 | 0.90 | 0.67 | 1.00 | 0.86 | Normal |
| 64 | 0.94 | 0.71 | 1.17 | 0.80 | 0.92 | 0.69 | 0.83 | 1.04 | 0.87 | 0.67 | 1.06 | 0.91 | Normal |
| 65 | 0.87 | 0.79 | 1.16 | 0.79 | 0.64 | 0.69 | 0.82 | 1.09 | 0.90 | 0.69 | 0.94 | 0.91 | Normal |
| 66 | 0.87 | 0.73 | 1.14 | 0.79 | 0.44 | 0.71 | 0.81 | 1.08 | 0.84 | 0.70 | 0.87 | 0.87 | Normal |
| 67 | 0.90 | 0.66 | 1.13 | 0.81 | 0.66 | 0.68 | 0.85 | 1.07 | 0.89 | 0.63 | 0.78 | 0.86 | Normal |
| 68 | 0.91 | 0.49 | 1.23 | 0.83 | 0.79 | 0.69 | 0.81 | 0.97 | 0.87 | 0.65 | 0.98 | 0.89 | Normal |
| 69 | 0.89 | 0.70 | 1.21 | 0.82 | 1.11 | 0.69 | 0.87 | 1.01 | 0.90 | 0.64 | 1.07 | 0.85 | Normal |
| 70 | 0.89 | 0.71 | 1.19 | 0.82 | 0.96 | 0.69 | 0.86 | 0.97 | 0.93 | 0.64 | 0.97 | 0.85 | Normal |
| 71 | 0.96 | 0.71 | 1.14 | 0.85 | 0.95 | 0.73 | 0.91 | 0.95 | 0.92 | 0.64 | 0.92 | 0.89 | Normal |
| 72 | 0.96 | 0.75 | 1.17 | 0.81 | 0.79 | 0.74 | 0.93 | 0.98 | 0.96 | 0.65 | 0.93 | 0.87 | Normal |
| 73 | 0.94 | 0.73 | 1.17 | 0.83 | 0.63 | 0.68 | 0.87 | 1.09 | 0.91 | 0.66 | 0.73 | 0.86 | Normal |
| 74 | 0.89 | 0.72 | 1.14 | 0.88 | 0.60 | 0.73 | 0.85 | 1.08 | 0.94 | 0.62 | 0.69 | 0.86 | Normal |
| 75 | 0.87 | 0.65 | 1.15 | 0.80 | 0.56 | 0.70 | 0.87 | 1.17 | 0.90 | 0.63 | 0.69 | 0.84 | Normal |
| 76 | 0.96 | 0.70 | 1.18 | 0.81 | 0.73 | 0.74 | 0.85 | 1.04 | 0.91 | 0.66 | 0.89 | 0.87 | Normal |
| 77 | 0.95 | 0.599 | 1.17 | 0.81 | 0.99 | 0.71 | 0.87 | 1.12 | 0.91 | 0.66 | 0.90 | 0.88 | Normal |
| 78 | 0.95 | 0.68 | 1.16 | 0.80 | 0.89 | 0.72 | 0.82 | 1.08 | 0.89 | 0.65 | 0.98 | 0.86 | Normal |
| 79 | 1.02 | 0.80 | 1.23 | 0.78 | 0.59 | 0.70 | 0.82 | 1.06 | 0.86 | 0.69 | 0.78 | 0.89 | Normal |
| 80 | 0.95 | 0.73 | 1.19 | 0.85 | 0.63 | 0.74 | 0.88 | 1.05 | 0.89 | 0.70 | 0.85 | 0.88 | Normal |
| 81 | 0.98 | 0.77 | 1.18 | 0.80 | 0.45 | 0.72 | 0.87 | 1.06 | 0.90 | 0.70 | 0.82 | 0.87 | Normal |
| 82 | 0.47 | 0.79 | 1.16 | 0.77 | 0.18 | 0.73 | 0.85 | 1.23 | 0.89 | 0.72 | 0.77 | 0.89 | Normal |
| 83 | 0.91 | 0.79 | 1.19 | 0.75 | 0.26 | 0.70 | 0.84 | 1.38 | 0.88 | 0.70 | 0.75 | 0.93 | Normal |
| 84 | 0.47 | 0.76 | 1.15 | 0.76 | 0.31 | 0.66 | 0.82 | 1.15 | 0.90 | 0.70 | 0.77 | 0.89 | Normal |
| 85 | 0.92 | 0.78 | 1.20 | 0.76 | 0.41 | 0.71 | 0.86 | 1.17 | 0.88 | 0.66 | 0.80 | 0.88 | Normal |
| 86 | 0.88 | 0.75 | 1.20 | 0.77 | 0.38 | 0.68 | 0.87 | 1.39 | 0.86 | 0.69 | 0.78 | 0.91 | Normal |
| 87 | 0.96 | 0.75 | 1.16 | 0.79 | 0.65 | 0.72 | 0.83 | 1.11 | 0.82 | 0.66 | 0.91 | 0.87 | Normal |
| 88 | 0.47 | 0.63 | 1.14 | 0.79 | 0.72 | 0.73 | 0.86 | 1.17 | 0.86 | 0.63 | 0.86 | 0.85 | Normal |
| 89 | 0.96 | 0.76 | 1.23 | 0.75 | 0.44 | 0.73 | 0.85 | 1.10 | 0.89 | 0.70 | 0.85 | 0.93 | Normal |
| 90 | 0.84 | 0.71 | 1.19 | 0.78 | 0.25 | 0.69 | 0.86 | 1.25 | 0.90 | 0.70 | 0.76 | 0.89 | Normal |
| 91 | 0.89 | 0.81 | 1.18 | 0.72 | 0.20 | 0.69 | 0.85 | 1.18 | 0.89 | 0.67 | 0.75 | 0.90 | Normal |
| 92 | 0.89 | 0.74 | 1.18 | 0.73 | 0.40 | 0.67 | 0.83 | 1.16 | 0.87 | 0.70 | 0.86 | 0.89 | Normal |
| 93 | 1.001 | 0.74 | 1.20 | 0.81 | 0.56 | 0.71 | 0.81 | 1.11 | 0.85 | 0.64 | 0.86 | 0.87 | Normal |
| 94 | 0.95 | 0.72 | 1.25 | 0.77 | 0.59 | 0.69 | 0.805 | 1.17 | 0.87 | 0.67 | 0.87 | 0.93 | Normal |
| 95 | 0.92 | 0.73 | 1.21 | 0.77 | 0.69 | 0.68 | 0.89 | 1.06 | 0.90 | 0.67 | 0.96 | 0.90 | Normal |
| 96 | 0.93 | 0.75 | 1.20 | 0.80 | 0.53 | 0.66 | 0.83 | 1.03 | 0.89 | 0.67 | 0.85 | 0.87 | Normal |
| 97 | 0.91 | 0.74 | 1.14 | 0.73 | 0.39 | 0.64 | 0.82 | 1.05 | 0.84 | 0.69 | 0.85 | 0.89 | Normal |
| 98 | 0.46 | 0.74 | 1.08 | 0.75 | 0.34 | 0.65 | 0.83 | 1.40 | 0.89 | 0.69 | 0.67 | 0.88 | Normal |
| 99 | 0.87 | 0.82 | 1.12 | 0.78 | 0.68 | 0.62 | 0.86 | 1.08 | 0.91 | 0.70 | 0.94 | 0.89 | Normal |
| 100 | 0.87 | 0.69 | 1.19 | 0.75 | 0.35 | 0.65 | 0.801 | 1.10 | 0.87 | 0.67 | 0.72 | 0.89 | Normal |
| 101 | 0.93 | 0.75 | 1.15 | 0.78 | 0.66 | 0.65 | 0.82 | 1.05 | 0.86 | 0.64 | 0.76 | 0.83 | Normal |
| 102 | 0.45 | 0.66 | 1.10 | 0.76 | 0.86 | 0.65 | 0.82 | 1.14 | 0.90 | 0.65 | 0.81 | 0.85 | Normal |
| 103 | 0.96 | 0.72 | 1.19 | 0.84 | 0.72 | 0.77 | 0.83 | 1.05 | 0.83 | 0.66 | 0.92 | 0.85 | Normal |
| 104 | 0.91 | 0.69 | 1.17 | 0.79 | 0.76 | 0.71 | 0.83 | 1.07 | 0.89 | 0.65 | 0.92 | 0.85 | Normal |
| 105 | 0.89 | 0.72 | 1.21 | 0.75 | 0.56 | 0.70 | 0.88 | 1.02 | 0.92 | 0.65 | 0.83 | 0.78 | Normal |
| 106 | 0.90 | 0.72 | 1.14 | 0.79 | 0.49 | 0.69 | 0.82 | 1.01 | 0.89 | 0.22 | 0.81 | 0.80 | Normal |
| 107 | 0.94 | 0.74 | 1.19 | 0.79 | 0.52 | 0.73 | 0.79 | 1.07 | 0.85 | 0.68 | 0.79 | 0.86 | Normal |
| 108 | 0.87 | 0.74 | 1.15 | 0.80 | 0.78 | 0.69 | 0.81 | 1.06 | 0.90 | 0.68 | 0.96 | 0.88 | Normal |
| 109 | 0.99 | 0.81 | 1.20 | 0.79 | 1.03 | 0.68 | 0.91 | 1.10 | 0.95 | 0.67 | 1.06 | 0.89 | Normal |
| 110 | 0.97 | 0.76 | 1.22 | 0.85 | 1.14 | 0.69 | 0.82 | 1.04 | 0.91 | 0.63 | 1.07 | 0.87 | Normal |
| 111 | 0.96 | 0.79 | 1.20 | 0.77 | 0.81 | 0.70 | 0.79 | 1.03 | 0.86 | 0.70 | 1.05 | 0.91 | Normal |
| 112 | 0.96 | 0.70 | 1.17 | 0.77 | 0.93 | 0.69 | 0.83 | 1.12 | 0.86 | 0.65 | 0.98 | 0.88 | Normal |
| 113 | 0.96 | 0.74 | 1.22 | 0.79 | 0.64 | 0.70 | 0.801 | 1.11 | 0.87 | 0.70 | 0.88 | 0.85 | Normal |
| 114 | 0.93 | 0.68 | 1.18 | 0.75 | 0.43 | 0.67 | 0.84 | 1.12 | 0.89 | 0.23 | 0.79 | 0.80 | Normal |
| 115 | 0.93 | 0.71 | 1.21 | 0.73 | 0.52 | 0.71 | 0.798 | 1.11 | 0.84 | 0.67 | 0.79 | 0.84 | Normal |
| 116 | 0.89 | 0.74 | 1.19 | 0.77 | 0.84 | 0.72 | 0.86 | 1.03 | 0.88 | 0.66 | 0.98 | 0.88 | Normal |
| 117 | 0.92 | 0.74 | 1.13 | 0.83 | 1.07 | 0.67 | 0.90 | 1.01 | 0.94 | 0.64 | 1.08 | 0.87 | Normal |
| 118 | 0.91 | 0.77 | 1.22 | 0.80 | 0.92 | 0.70 | 0.84 | 1.02 | 0.92 | 0.64 | 0.95 | 0.89 | Normal |
| 119 | 0.98 | 0.78 | 1.21 | 0.83 | 0.74 | 0.71 | 0.84 | 1.06 | 0.93 | 0.61 | 0.84 | 0.84 | Normal |
| 120 | 0.95 | 0.73 | 1.22 | 0.82 | 0.76 | 0.72 | 0.83 | 1.09 | 0.95 | 0.68 | 0.84 | 0.88 | Normal |
| 121 | 0.92 | 0.75 | 1.18 | 0.78 | 0.57 | 0.70 | 0.86 | 1.24 | 0.93 | 0.68 | 0.81 | 0.88 | Normal |
| 122 | 0.93 | 0.72 | 1.16 | 0.83 | 0.42 | 0.69 | 0.82 | 1.13 | 0.91 | 0.24 | 0.72 | 0.85 | Normal |
| 123 | 0.92 | 0.74 | 1.14 | 0.76 | 0.48 | 0.70 | 0.85 | 1.16 | 0.91 | 0.66 | 0.76 | 0.88 | Normal |
| 124 | 0.93 | 0.81 | 1.17 | 0.76 | 0.66 | 0.67 | 0.86 | 1.11 | 0.90 | 0.69 | 0.88 | 0.84 | Normal |
| 125 | 0.93 | 0.77 | 1.17 | 0.79 | 0.86 | 0.68 | 0.89 | 1.19 | 0.86 | 0.65 | 0.96 | 0.87 | Normal |
| 126 | 0.998 | 0.77 | 1.22 | 0.82 | 0.87 | 0.73 | 0.801 | 1.15 | 0.87 | 0.62 | 0.92 | 0.89 | Normal |
| 127 | 0.98 | 0.75 | 1.11 | 0.80 | 0.59 | 0.72 | 0.83 | 1.11 | 0.86 | 0.68 | 0.88 | 0.90 | Normal |
| 128 | 0.90 | 0.75 | 1.16 | 0.77 | 0.87 | 0.68 | 0.88 | 1.07 | 0.90 | 0.69 | 0.93 | 0.88 | Normal |
| 129 | 0.93 | 0.76 | 1.21 | 0.80 | 0.64 | 0.68 | 0.82 | 1.12 | 0.89 | 0.68 | 0.94 | 0.87 | Normal |
| 130 | 0.88 | 0.77 | 1.16 | 0.77 | 0.57 | 0.68 | 0.86 | 1.05 | 0.89 | 0.68 | 0.89 | 0.88 | Normal |
| 131 | 0.90 | 0.76 | 1.21 | 0.78 | 0.54 | 0.68 | 0.89 | 1.09 | 0.90 | 0.67 | 0.83 | 0.92 | Normal |
| 132 | 0.93 | 0.75 | 1.22 | 0.77 | 0.65 | 0.69 | 0.84 | 1.05 | 0.94 | 0.65 | 0.95 | 0.88 | Normal |
| 133 | 0.88 | 0.79 | 1.16 | 0.75 | 0.82 | 0.68 | 0.82 | 1.06 | 0.85 | 0.69 | 1.01 | 0.89 | Normal |
| 134 | 0.94 | 0.73 | 1.15 | 0.79 | 0.82 | 0.71 | 0.83 | 1.13 | 0.89 | 0.68 | 0.92 | 0.88 | Normal |
| 135 | 0.93 | 0.76 | 1.18 | 0.78 | 0.61 | 0.70 | 0.85 | 1.16 | 0.84 | 0.71 | 0.93 | 0.89 | Normal |
| 136 | 0.89 | 0.76 | 1.15 | 0.76 | 0.74 | 0.69 | 0.799 | 1.11 | 0.801 | 0.72 | 0.94 | 0.88 | Normal |
| 137 | 0.90 | 0.76 | 1.21 | 0.82 | 0.71 | 0.68 | 0.88 | 1.21 | 0.88 | 0.68 | 0.92 | 0.91 | Normal |
| 138 | 0.89 | 0.76 | 1.19 | 0.77 | 0.42 | 0.67 | 0.84 | 1.11 | 0.84 | 0.68 | 0.85 | 0.85 | Normal |
| 139 | 0.86 | 0.80 | 1.17 | 0.76 | 0.39 | 0.68 | 0.82 | 1.19 | 0.88 | 0.73 | 0.85 | 0.91 | Normal |
| 140 | 0.90 | 0.73 | 1.16 | 0.74 | 0.57 | 0.67 | 0.799 | 1.03 | 0.84 | 0.69 | 0.91 | 0.88 | Normal |
| 141 | 0.91 | 0.71 | 1.21 | 0.77 | 0.94 | 0.68 | 0.81 | 1.10 | 0.87 | 0.64 | 0.86 | 0.85 | Normal |
| 142 | 0.85 | 0.78 | 1.19 | 0.75 | 0.91 | 0.73 | 0.81 | 1.18 | 0.91 | 0.67 | 0.97 | 0.89 | Normal |
| 143 | 0.98 | 0.77 | 1.15 | 0.80 | 0.68 | 0.69 | 0.81 | 1.09 | 0.87 | 0.62 | 0.92 | 0.86 | Normal |
| 144 | 0.86 | 0.79 | 1.18 | 0.79 | 0.94 | 0.65 | 0.83 | 1.19 | 0.85 | 0.71 | 1.04 | 0.88 | Normal |
| 145 | 0.91 | 0.76 | 1.17 | 0.76 | 0.58 | 0.68 | 0.81 | 1.16 | 0.85 | 0.69 | 0.94 | 0.88 | Normal |
| 146 | 0.87 | 0.69 | 1.21 | 0.80 | 0.60 | 0.69 | 0.82 | 1.10 | 0.85 | 0.71 | 0.75 | 0.89 | Normal |
| 147 | 0.88 | 0.71 | 1.17 | 0.76 | 0.49 | 0.70 | 0.81 | 1.09 | 0.89 | 0.67 | 0.79 | 0.89 | Normal |
| 148 | 0.83 | 0.78 | 1.13 | 0.82 | 0.63 | 0.67 | 0.82 | 1.10 | 0.84 | 0.68 | 0.83 | 0.89 | Normal |
| 149 | 0.94 | 0.68 | 1.21 | 0.78 | 0.62 | 0.64 | 0.83 | 1.13 | 0.87 | 0.65 | 0.79 | 0.86 | Normal |
| 150 | 0.89 | 0.81 | 1.15 | 0.77 | 0.72 | 0.67 | 0.86 | 1.16 | 0.93 | 0.69 | 0.85 | 0.90 | Normal |
| 151 | 0.99 | 0.73 | 1.19 | 0.76 | 0.65 | 0.65 | 0.85 | 1.09 | 0.88 | 0.72 | 0.97 | 0.90 | Normal |
| 152 | 0.90 | 0.83 | 1.19 | 0.79 | 0.76 | 0.70 | 0.87 | 1.17 | 0.93 | 0.71 | 0.95 | 0.97 | Normal |
| 153 | 0.89 | 0.78 | 1.13 | 0.78 | 0.49 | 0.67 | 0.83 | 1.17 | 0.87 | 0.69 | 0.85 | 0.89 | Normal |
| 154 | 0.93 | 0.83 | 1.15 | 0.78 | 0.30 | 0.74 | 0.87 | 1.12 | 0.90 | 0.70 | 0.82 | 0.92 | Normal |
| 155 | 0.90 | 0.85 | 1.11 | 0.77 | 0.28 | 0.67 | 0.81 | 1.15 | 0.89 | 0.69 | 0.80 | 0.92 | Normal |
| 156 | 0.89 | 0.82 | 1.15 | 0.74 | 0.36 | 0.66 | 0.85 | 1.06 | 0.90 | 0.72 | 0.83 | 0.95 | Normal |
| 157 | 0.94 | 0.78 | 1.18 | 0.78 | 0.38 | 0.71 | 0.85 | 1.17 | 0.94 | 0.70 | 0.84 | 0.91 | Normal |
| 158 | 0.93 | 0.79 | 1.16 | 0.77 | 0.48 | 0.69 | 0.84 | 1.17 | 0.90 | 0.72 | 0.82 | 0.92 | Normal |
| 159 | 0.78 | 0.75 | 1.18 | 0.79 | 0.56 | 0.67 | 0.87 | 1.18 | 0.90 | 0.69 | 0.899 | 0.88 | Normal |
| 160 | 0.93 | 0.71 | 1.17 | 0.73 | 0.63 | 0.64 | 0.86 | 1.15 | 0.86 | 0.72 | 0.96 | 0.94 | Normal |
| 161 | 0.91 | 0.83 | 1.15 | 0.76 | 0.45 | 0.70 | 0.84 | 1.15 | 0.85 | 0.68 | 0.89 | 0.94 | Normal |
| 162 | 0.88 | 0.81 | 1.19 | 0.75 | 0.28 | 0.73 | 0.88 | 1.19 | 0.91 | 0.72 | 0.85 | 0.94 | Normal |
| 163 | 0.97 | 0.82 | 1.16 | 0.78 | 0.28 | 0.74 | 0.86 | 1.16 | 0.91 | 0.69 | 0.82 | 0.87 | Normal |
| 164 | 0.83 | 0.79 | 1.18 | 0.73 | 0.46 | 0.64 | 0.800 | 1.22 | 0.87 | 0.70 | 0.85 | 0.92 | Normal |
| 165 | 0.82 | 0.81 | 1.26 | 0.76 | 0.58 | 0.68 | 0.83 | 1.17 | 0.89 | 0.67 | 0.903 | 0.92 | Normal |
| 166 | 0.92 | 0.78 | 1.19 | 0.79 | 0.57 | 0.69 | 0.87 | 1.16 | 0.92 | 0.65 | 0.89 | 0.90 | Normal |
| 167 | 0.92 | 0.73 | 1.20 | 0.76 | 0.68 | 0.65 | 0.86 | 1.14 | 0.88 | 0.65 | 0.88 | 0.87 | Normal |
| 168 | 0.96 | 0.72 | 1.17 | 0.79 | 0.59 | 0.66 | 0.89 | 1.07 | 0.88 | 0.67 | 0.94 | 0.91 | Normal |
| 169 | 0.94 | 0.84 | 1.15 | 0.78 | 0.42 | 0.63 | 0.82 | 1.09 | 0.85 | 0.67 | 0.84 | 0.88 | Normal |
| 170 | 0.91 | 0.80 | 1.15 | 0.76 | 0.42 | 0.64 | 0.82 | 1.14 | 0.90 | 0.71 | 0.76 | 0.95 | Normal |
| 171 | 0.86 | 0.82 | 1.10 | 0.74 | 0.31 | 0.67 | 0.804 | 1.18 | 0.86 | 0.69 | 0.84 | 0.86 | Normal |
| 172 | 0.77 | 0.76 | 1.15 | 0.73 | 0.43 | 0.63 | 0.86 | 1.07 | 0.87 | 0.67 | 0.78 | 0.92 | Normal |
| 173 | 0.90 | 0.73 | 1.15 | 0.74 | 0.60 | 0.61 | 0.82 | 1.09 | 0.85 | 0.67 | 0.76 | 0.87 | Normal |
